# Genetic predisposition to advanced biological ageing increases risk for childhood-onset recurrent major depressive disorder in a large UK sample

**DOI:** 10.1016/j.jad.2017.01.017

**Published:** 2017-04-15

**Authors:** Julia E. Michalek, Agnieszka Kepa, John Vincent, Souci Frissa, Laura Goodwin, Matthew Hotopf, Stephani L. Hatch, Gerome Breen, Timothy R. Powell

**Affiliations:** a King's College London, MRC Social, Genetic and Developmental Psychiatry Centre, Institute of Psychiatry, Psychology & Neuroscience, London, UK; b National Institute for Health Research Biomedical Research Centre for Mental Health, Institute of Psychiatry, Psychology and Neuroscience at the Maudsley Hospital and King's College London, UK; c King's College London, Health Service & Population Research, Institute of Psychiatry, Psychology & Neuroscience, London, UK; d King's College London, Psychological Medicine, Institute of Psychiatry, Psychology & Neuroscience, London, UK; e University of Liverpool, Department of Psychological Sciences, Liverpool, UK

## Abstract

**Background:**

Previous studies have revealed increased biological ageing amongst major depressive disorder (MDD) patients, as assayed by shorter leukocyte telomere lengths (TL). Stressors such as childhood maltreatment are more common amongst MDD patients, and it has been suggested that this might contribute to shorter TL present amongst patients. However, to our knowledge, no study has yet tested for reverse causality, i.e. whether a genetic predisposition to shorter TL might predispose to MDD or an earlier onset of MDD.

**Methods:**

This study used a Mendelian randomisation design to investigate if shortened TL might increase risk for recurrent MDD in a relatively large UK sample (1628 MDD cases, 1140 controls). To achieve this, we used a subset of our sample, for which TL data was available, to identify a suitable instrumental variable. We performed single nucleotide polymorphism (SNP) genotyping on rs10936599, a SNP upstream of telomerase RNA component (*TERC*), and rs2736100, a SNP within telomerase reverse transcriptase (*hTERT*), and attempted to replicate findings which identified these SNPs as predictors of TL. After which, we performed regressions to test if genetic risk for shortened TL increased risk for MDD, childhood-onset MDD or childhood/adolescent-onset MDD.

**Results:**

T-carriers of rs10936599 demonstrated shorter TL compared to CC-carriers (p≤0.05; 3% of variance explained) and was subsequently used as our instrumental variable. We found that the T-allele of rs10936599 predicted increased risk for childhood-onset MDD relative to controls (p≤0.05), and increased risk for childhood-onset MDD relative to adult-onset MDD cases (p≤0.001), but rs10936599 did not predict adult-onset MDD risk.

**Limitations:**

Limitations include a relatively small sample of early-onset cases, and the fact that age-of-onset was ascertained by retrospective recall.

**Conclusion:**

Genetic predisposition to advanced biological ageing, as assayed using rs10936599, predicted a small, but significant, increased risk for childhood-onset recurrent MDD. Genetic predisposition to advanced biological ageing may be one factor driving previously reported associations (or lack of associations) between shorter TL and MDD. Our results also suggest that the telomerase enzyme may act as a potentially important drug target for the prevention of childhood-onset MDD, at least in a subset of cases. Future studies should attempt to replicate our findings in a larger cohort.

## Highlights

• T-carriers of a single nucleotide polymorphism within the telomerase RNA component gene (rs10936599) predicts shorter telomere lengths, and therefore advanced biological ageing.
• T-carriers of rs10936599 are also significantly more likely to be diagnosed with childhood-onset recurrent major depression.
• Our study suggests that a genetic predisposition to advanced biological ageing increases risk for early onset recurrent major depression.

## 1 Introduction

Telomeres are capping structures of tandem TTAGGG nucleotide repeats found at the end of chromosomes (Eitan et al., 2014). During each cell division, the ends of chromosomes shorten as part of a natural consequence of replication (Aubert and Lansdorp, 2008). Telomeres function as sacrificial, non-coding DNA buffers, which degrade instead of inward, coding DNA regions (Allsopp et al., 1995). Eventually, in cells which have undergone many divisions, telomeres become so short that the coding DNA regions within the chromosome are no longer protected, and their degradation triggers the end of that cell's ability to replicate (Eitan et al., 2014). Telomere length (TL) subsequently acts as marker for ‘cellular age’ or ‘biological age’; with shortened telomeres representing older cells, and commonly, older individuals (Benetos et al., 2001). However, unlike chronological age, biological ageing can be moderated by environmental and genetic factors (e.g. Tyrka et al., 2010, Codd et al., 2013), meaning two unrelated individuals of the same chronological age, may not be the same age biologically. Shortened leukocyte TL, relative to one's age, has been associated with an increased risk to various diseases, generally poorer physical and psychiatric health, and higher mortality (Simon et al., 2006, Fitzpatrick et al., 2007, Kao et al., 2008, Yu et al., 2008, Okereke et al., 2012, Lindqvist et al., 2015, Rode et al., 2015, Darrow et al., 2016).

Evidence suggests that an increased stress hormone response (cortisol levels), oxidative stress, and immuno-inflammatory activation, could be responsible for some of these inter-individual differences in TL observed within the population (von Zglinicki, 2002, Jurk et al., 2014, Gotlib et al., 2015). A disease which has been linked to all three of these telomere-eroding factors, is major depressive disorder (MDD; Cowen, 2002; Michel et al., 2012; Martin et al., 2015). Indeed, most previous studies (e.g. Simon et al., 2006; Lung et al., 2007; Elvsåshagen et al., 2011; Hoen et al., 2011; Garcia-Rizo et al., 2013; Verhoeven et al., 2014) but not all (e.g. Wolkowitz et al., 2011; Teyssier et al., 2012; Needham et al., 2015; Schaakxs et al., 2015), have revealed shortened leukocyte TL amongst MDD patients with some studies suggesting that shortened TLs may be observed most pervasively in recurrent depressed cases only (e.g. Elvsåshagen et al., 2011). Interestingly, a history of childhood maltreatment (a risk factor for MDD) also predicts shortened TL in adulthood (Tyrka et al., 2010, O'Donovan et al., 2011; Kananen et al., 2010; Shalev et al., 2014). This has generated hypotheses which suggest that stress may simultaneously precipitate risk for MDD, and an advancement in telomere shortening; contributing to the increased risk of comorbid ageing-related disorders present amongst MDD patients; including cardiovascular disease, obesity, and type-2 diabetes (Zhang et al., 2014). Consequently, it's been hypothesized that negative environmental factors, such as stress, are primarily responsible for shortened leukocyte TL present amongst MDD patients. However, few reports have considered the possibility of reverse causality, i.e. whether a predisposition to advanced biological ageing (i.e. genetic factors) may also predispose an individual to MDD.

A recent genome-wide association study (GWAS) revealed single nucleotide polymorphisms (SNPs) predictive of relative TL; with the two most significant SNPs rs10936599 and rs2736100, located upstream or within the telomerase encoding genes telomerase RNA component (*TERC*) and telomerase reverse transcriptase (*hTERT*), respectively (Codd et al., 2013). These SNPs are hypothesized to affect the functionality of telomerase, an enzyme with the ability to reverse telomere shortening, by adding TTAGGG sequences to the existing telomere ends (Codd et al., 2013). Thus, SNPs coding for this enzyme represent functionally discrete factors with pervasive effects on long-term TL maintenance. They also represent a means by which we can test if inherent genetic factors influencing TL maintenance predict risk for MDD.

Mendelian randomisation is an ‘instrumental variable analysis’ and is the formal term used to describe a situation where we test whether genetic factors (a ‘instrumental variable’) contributing to a biological factor correlating with a disease (e.g. shortened telomere lengths) directly predicts the disease itself (e.g. MDD; Sheehan et al., 2008; Smith and Hermani, 2014). If it does, this would suggest that the biological correlate may be involved in causing the disease, but if it does not, it may indicate it is an effect of having the disease, or that an independent factor impacts upon both the biological correlate and the disease.

Within this study we adopted a Mendelian randomisation design to investigate whether a genetic predisposition to advanced biological ageing (via rs10936599, rs2736100) predicted an increased risk of recurrent MDD in a large UK sample. As MDD is generally an adult-onset disorder (Kessler et al., 2001) and has been repeatedly associated with biological ageing and risk for ageing-related disease (Zhang et al., 2014), we also tested whether a genetic predisposition to advanced biological ageing might shorten the time it takes for MDD to present itself, i.e. increase risk for childhood (≤12 years old) or childhood/adolescent-onset (≤17 years old) MDD. These earlier-onset time points were chosen because they represent key, well-characterised, developmental milestones and times during which there are increased rates of cellular division, relative to adulthood. Therefore, phenotypes which result from particular cell populations having a limited proliferative potential (as a result of advanced biological ageing), may begin to precipitate at these earlier time points.

To achieve our aims effectively, we first attempted to replicate Codd and colleagues’ findings using relative TL data from an independent cohort (and a subset of our genetic cohort), and to determine the best genetic model and/or combination of the two SNPs (rs10936599 and rs2736100) to use as our ‘instrumental variable’. Secondly, using a large UK cohort of 1628 recurrent MDD cases and 1140 control subjects, we tested whether the relative frequency of risk alleles for shorter TL was greater amongst MDD cases, or early-onset MDD cases.

## 2 Methods

### 2.1 Subject information

Recurrent MDD cases were recruited from the UK component of RADIANT, described previously (Lewis et al., 2010). Controls (n=1140) were recruited from the Depression case-control study (n=1040; Cohen-Woods et al., 2009), and from the South East London Community Health Study (SELCoH, n=100; Hatch et al., 2011). For a full break down, see Table 1.

**Table 1:** Descriptive statistics of case/control subjects within study; including total number of participants, mean age at interview (with Standard Deviation) and numbers within each gender.

**Study**	**N**	**Age**	**Males**	**Females**
DeCC cases	1248	47 (SD = 12.34)	380	868
GENDEP cases	83	46 (SD = 12.3)	29	54
DENT cases	297	46 (SD = 10.9)	71	226
*Total Cases*	1628	46.26 (SD =10.8)	480	1148
DeCC controls	1040	45 (SD = 9.8)	429	611
SELCoH controls	100	51 (SD = 16.9)	49	51
*Total Controls*	1140	46 (SD = 10.8)	481	669
**Total**	2768	46 (SD = 11.6)	958	1811

### 2.2 Recurrent MDD cases

RADIANT is an umbrella term for three studies which sought to understand genetic risk for MDD and factors affecting response to treatment; this comprised of the Depression Network (DeNT) study (Farmer et al., 2004), the Depression Case-Control (DeCC) study (Cohen-Woods et al., 2009) and the Genome-Based Therapeutic Drugs for Depression (GENDEP) study (Uher et al., 2009). Within these multi-centre clinical studies, we selected only those recruited from the UK who had at least two episodes of major depression of at least moderate severity, in order to create a homogeneous sample. Diagnosis of MDD was ascertained using the Schedules for Clinical Assessment in Neuropsychiatry (SCAN) interview in all three studies (Wing et al., 1998), which was used to generate International Classification of Diseases, 10th edition (ICD-10; World Health Organisation, 1992), and the Diagnostic and Statistical Manual of Mental Disorders, revised third edition (DSM-III-R; American Psychiatric Association, 1987), diagnoses. People who had ever fulfilled criteria of intravenous substance dependence, substance-induced mood disorder, schizophrenia or bipolar disorder were excluded from all three studies. Information on the age of onset of MDD and number of episodes were obtained by questionnaires and clinical interviews. In total there were 206 childhood-onset cases, and 518 childhood/adolescent-onset cases. All participants were of White European ancestry.

### 2.3 Control subjects

The majority of controls were derived from those recruited as part of DeCC, a subset of RADIANT. Subjects were screened for lifetime absence of any psychiatric disorder using a modified version of the Past History Schedule (McGuffin et al., 1986). Participants were excluded if they, or a first-degree relative, ever fulfilled the criteria for MDD or any other psychiatric disorder. We also included a subset of SELCoH subjects as controls, genotyped within the current study. SELCoH is a population study in London, UK, investigating community health (Hatch et al., 2011). It's an on-going study, in which phenotypic information has been collected over three phases over an eight-year period, with blood being collected in the third phase. Control subjects were identified as those with no depression symptoms on any of the three assessment phases, as measured using the Clinical Interview Schedule-Revised (Lewis et al., 1992), and no previous history of a depressive disorder as ascertained using a self-report questionnaire. 77% of the SELCoH controls showed no other psychiatric symptoms outside of MDD in all three phases (neurotic disorders, anxiety disorders, obsessive compulsive disorder, phobias, panic disorder); with 93% having no psychiatric symptoms at the time of blood collection. Therefore, 97.8% of our total control group contained individuals with no lifetime history of any psychiatric symptoms. All participants were of White European ancestry.

### 2.4 Ethics

The SELCoH study received approval from the King's College London research ethics committee, reference PNM/12/13–152. The RADIANT studies were approved by the Joint South London and Maudsley NHS Trust Institute of Psychiatry Research Ethics Committee. Informed written consent was obtained from all the participants at the time of sample collection.

### 2.5 Validating our instrumental variable

In order to perform Mendelian randomisation we first attempted to validate whether rs10936599 or rs2736100 predicted relative TL in our sample. We used TL data that was collected as part of a different study (n=180; Vincent et al., under submission). Briefly, subjects in this subsample were chosen based on: (i) availability of leukocyte DNA samples, (ii) participants being White and from the UK (due to population differences in TL), (iii) availability of information on depressive disorder case/control status and childhood maltreatment.

We attempted to validate our instrumental variables using data generated from all 180 subjects, with 125 of these subjects (SELCoH controls and recurrent MDD patients from DeCC only) also included in our main analysis investigating genetic risk to biological ageing and its relationship to recurrent MDD.

### 2.6 Relative telomere length in SELCoH and DeCC subset

Briefly, TL was quantified in our sample subset using the output from two separate quantitative real-time polymerase chain reactions (qPCRs). The first qPCR assays the telomere repeat region (TTAGGG), and the second qPCR assays a single copy gene (albumin). The ratio between the telomere repeat region and the single copy gene was calculated to determine relative TL. Relative TL was then log transformed and adjusted for the confounding effects of age, gender and study by taking the standardized residuals. Previous work on this data set found no confounding effects of body mass index, smoking habits, antidepressant use, drug dependency, drug use, other medication use, or comorbid diseases, on relative telomere length (Vincent et al., under submission). The output was used to test the effect of SNPs on adjusted log(relative TL). For full details, see Supplementary Information.

### 2.7 SNP genotyping in the SELCoH sample

Genotyping of 155 subjects within SELCoH was performed within the current study. Genomic DNA within SELCoH was extracted from blood using standard extraction methods (Freeman et al., 2003). One negative (no template) control sample for each gene (2.6 μl RNase-free water) was included to confirm absence of nucleic acid contamination. SNP genotyping was assayed using a Taqman SNP genotyping assay (Thermo Fisher Scientific, Massachusetts, United States). Each reaction mix consisted of 2.5 μL 2x Taqman Genotyping Mastermix (Thermo Fisher Scientific), 0.125 μL 40x Taqman Genotyping Assay Mix (Thermo Fisher Scientific), and 10 ng DNA. rs10936599 (Cat. # 4351379) and rs2736100 (Cat. # 4351379) SNP genotyping assays contained allele-specific primers which were tagged with either FAM or VIC labelled probes, Table 2. The differences in fluorescence emission allows for both alleles to be detected simultaneously within a single well.

**Table 2:** Details of rs10936599 and rs2736100 including their location, the genomic region assayed by VIC and FAM probes, and minor and major allele frequencies.

**rs10936599 (*****TERC*****)**	
Location	Chr.3;169492101
Context Sequence (VIC/FAM)	ATATCAAAATGCAGTATTCGCACCA**[C/T]**TGTGAGCACCTTTTAGAGAGACTGA
Minor Allele Frequency	T = 0.27
Major Allele Frequency	C = 0.73

**rs2736100 (*****hTERT*****)**	
Location	Chr.5;1286516
Context Sequence (VIC/FAM)	GAAAAGCAGGGCGGGGGCAAAGCTA**[A/C]**AGAAACACTCAACACGGAAAACAAT
Minor Allele Frequency	A = 0.47
Major Allele Frequency	C = 0.53

The polymerase chain reaction was performed using the ABI Prism 7900HT Sequence Detection System (Applied Biosystems, Massachusetts, USA) and an allelic discrimination analysis using SDS 2.3 (Applied Biosystems) was used to determine genotypes within each of the 155 samples included on a 384-well plate, following the standard manufacturer's protocol.

### 2.8 Already available genotype data from RADIANT

SNP data from RADIANT was already available. Genomic DNA within RADIANT was extracted from bloods and cheek swabs collected as described previously (Freeman et al., 2003). DNA samples were then sent to the Centre National de Genotypage (Evry Cedex, France) and were genotyped using the Illumina Human610-Quad bead chip (Illumina, Inc., San Diego, CA, USA). Genotype data for single nucleotide polymorphisms (SNPs) under investigation in this study were extracted using PLINK (Purcell et al., 2007).

### 2.9 Statistical analysis

(i) Validation of our instrumental variable: First, we performed a Chi-Square test to ensure the 180 subjects with TL data were representative of the population, and that the relative frequency of alleles did not deviate from Hardy-Weinberg equilibrium. Subsequently, we performed univariate linear regressions to determine the effect of rs10936599 and rs2736100 on adjusted log(relative TL) as part of additive, dominant and recessive models.
(ii) Case-control comparison: Again, we tested whether the relative frequency of alleles in this larger cohort deviated from Hardy-Weinberg equilibrium. We then performed a generalized linear model with the binomial distribution and specified identity link function (Wacholder 1986) in order to establish risk difference (RD), as previously done in Fisher et al. (2013). We included MDD case/control status as the outcome variable, with SNP(s) predicting telomere length, and gender, included as independent factors.
(iii) Age of onset comparison: We tested whether SNP(s) predicting TL may predict early onset MDD. We tested the effect of SNP(s) on childhood-onset MDD (≤12 years old), child/adolescent-onset MDD (≤17 years old) and also for comparison, adult-onset MDD (≥18 years old). To achieve this we performed the same generalized linear model as above but with a subset of cases based on age-of-onset, and all controls included. We also compared early-onset cases with adult-onset MDD cases. The false discovery rate (FDR) method of multiple testing correction was used to determine true associations in analyses (ii) and (iii), with a q value threshold of q<0.05.

## 3 Results

### 3.1 Validation of instrumental variable

There was a 100% call rate for both rs10936599 and rs2736100 for all 155 DNA samples which underwent SNP genotyping, and there was no amplification in the negative control. In the total sample of 180, for which there was corresponding TL data, neither rs10936599 [CC=98, CT=74, TT=8; χ^2^=1.67, p=0.196], or rs2736100 [AA=44, AC=92, CC=44; χ^2^=0.09, p=0.764], deviated from Hardy-Weinberg equilibrium.

**Fig. 1:**
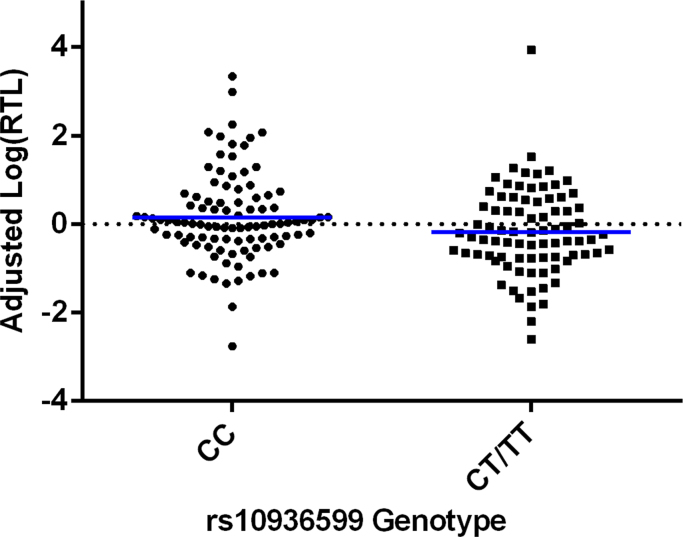
A plot showing the effect of rs10936599 on adjusted log(relative TL). Genotypes with one or two risk alleles (TC/TT) are significantly associated with shorter adjusted telomere length relative to genotypes with no risk allele (C/C), p<0.05.

Subsequently, we used a series of univariate linear regressions to determine which allelic combination explained the greatest variance in adjusted log(relative TL), and thus which allelic combination should be utilized as our ‘instrumental variable’. In the case of rs2736100, the A-allele represents the risk SNP for shortened TL, in the case of rs10936599, the T-allele represents the risk SNP for shortened TL (Codd et al., 2013). Our results are summarized in Table 3. We found that the presence/absence of the T-allele (rs10936599; dominant model) was the strongest predictor of adjusted log(relative TL), explaining 3% of the variance, Fig. 1.

**Table 3:** Results from univariate linear regressions investigating the allelic combinations of rs2736100 and rs10936599 as predictors of adjusted log(relative TL) in 180 UK subjects. rs10936599 genotype was found to significantly predict adjusted log(relative TL) as part of both an additive and dominant model. The dominant model was the most significant predictor and explained the most variance, so was selected as our ‘instrumental variable’.

**SNP**	**Model Tested**	**F**	***P*****value**	**Variance Explained**
rs2736100	Additive (CC=0, AC=1, AA=2)	2.960E-04	0.986	0.000
rs2736100	Dominant (CC=0, AC/AA=1)	0.370	0.544	0.002
rs2736100	Recessive (AA=0, AC/CC=1)	0.416	0.520	0.002
rs10936599	Additive (CC=0, TC=1, TT=2)	4.884	0.028	0.027
rs10936599	Dominant (CC=0, TC/TT=1)	5.237	0.023*	**0.029**
rs10936599	Recessive (TT=0, TC/CC=1)	1.513	0.220	0.009
rs2736100 + rs10936599	Additive (CC=0, AC=1, AA=2; CC=0, TC=1, TT=2)	1.949	0.164	0.011

### 3.2 rs10936599 as a predictor of recurrent MDD and age of onset comparison

In addition to utilizing data from our subset with corresponding TL data, we also used previously collected genotype data from RADIANT for subsequent analyses. To ensure this combined UK sample remained representative of the general population, we again checked if rs10936599 fell within Hardy-Weinberg equilibrium. We found no deviation from Hardy-Weinberg equilibrium in the total sample (CC=1577; CT=1012; TT=179; χ^**2**^=0.900; p=0.343).

**Fig. 2:**
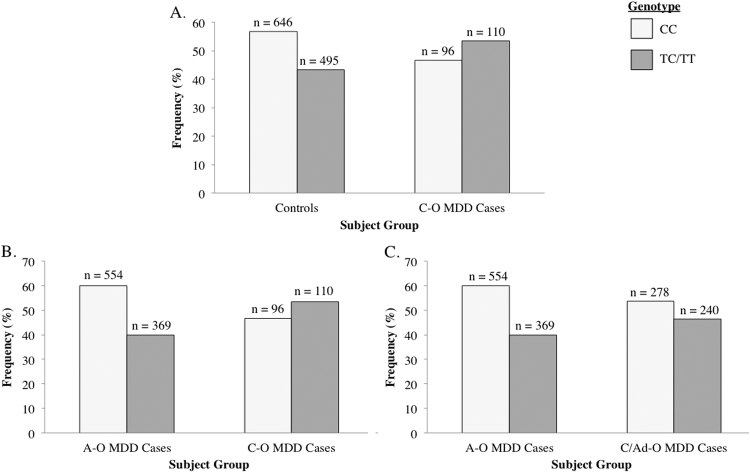
The relative frequency (%) of CC versus TC/TT carriers for rs10936599 amongst: (A) controls and childhood-onset (C-O) recurrent MDD cases; (B) adult-onset (A-O) recurrent MDD cases and childhood-onset (C-O) recurrent MDD cases; (C) adult-onset (A-O) recurrent MDD cases and childhood/adolescent-onset (C/Ad-O) recurrent MDD cases. Absolute numbers in each group are shown on top of each bar. There were significantly higher numbers of T-allele carriers amongst childhood-onset or childhood/adolescent recurrent MDD cases in all groups (p<0.05).

Generalized linear models revealed no effect of rs10936599 genotype (TT/CT versus CC) on general MDD case/control status (p=0.700), or adult-onset MDD (p=0.106), Table 4. To investigate if rs10936599 might predict earlier onset MDD, we investigated whether rs10936599 predicted childhood-onset MDD (≤12 years old) as part of a case-control comparison, and as part of a childhood-onset MDD case versus adult onset (≥18 years old) MDD within-case comparison. Similarly, we did the same analyses for childhood + adolescent onset MDD cases (≤17 years old). rs10936599 significantly predicted childhood onset MDD both as part of a case-control comparison (p=0.012; Fig. 2), and as part of a childhood-onset versus adult-onset MDD within-case comparison (p=0.001; Fig. 2). To a lesser extent, rs10936599 also predicted childhood/adolescent-onset MDD relative to adult-onset MDD cases (p=0.02; Fig. 2). The FDR method of multiple testing correction was used, and confirmed that all three effects remained significant at a q threshold of q<0.05.

**Table 4:** The effects of carrying the T-allele of rs10936599 on risk for recurrent MDD, childhood-onset recurrent MDD, or childhood/adolescent onset recurrent MDD. Results include the sex adjusted relative risk differences, p-values, confidence intervals and FDR-corrected q-values. * indicate significant effects (q<0.05).

**rs10936599 effect on MDD**	**Adjusted Relative Risk Difference**	***P*****value**	**95% Confidence Intervals**		**Q-value**
All MDD Cases v Controls	−0.007	0.700	−0.044	0.029	0.700
Adult onset MDD v Controls	−0.356	0.106	−0.079	0.007	0.159
Childhood/adolescent onset MDD v Controls	0.023	0.296	−0.021	0.068	0.355
Childhood onset MDD v Controls	0.048	0.012*	0.011	0.087	0.036
Childhood/adolescent onset MDD v Adult onset MDD	0.060	0.020*	0.009	0.110	0.040
Childhood onset MDD v Adult onset MDD Cases	0.082	0.001*	0.035	0.128	0.006

## 4 Discussion

The aim of this study was to investigate the effect of rs10936599 and rs2736100 on leukocyte TL and subsequently the impact of genetic risk for shorter TL on risk for recurrent MDD status, childhood-onset recurrent MDD, and childhood/adolescent-onset recurrent MDD. First, our results revealed that the T-allele of rs10936599 was the best predictor of shortened TL in a subset of our sample, Fig. 1, Table 3. The T-allele was the most significant predictor of shortened telomere length in an independent GWAS consisting of the largest sample to-date (Codd et al., 2013), and thus, we felt confident in using rs10936599 as our instrumental variable predicting increased risk for biological ageing.

We subsequently performed Mendelian randomisation to probe if genetic predisposition to advanced biological ageing (T-carriers of rs10936599) predicted risk for recurrent MDD. rs10936599 did not predict risk for recurrent MDD in our general case-control comparison, nor to adult-onset recurrent MDD. However, our study revealed that a genetic predisposition to advanced biological ageing may increase risk for early-onset MDD. We found a small but statistically significant increased risk to childhood-onset recurrent MDD relative to controls, and a significant increased relative risk to recurrent childhood-onset MDD relative to recurrent adult-onset cases amongst T-allele carriers of rs10936599, Fig. 2, Table 4. To a lesser extent we also found an increased risk for recurrent childhood/adolescent-onset MDD relative to recurrent adult-onset MDD, Fig. 2, Table 4.

Childhood and adolescent MDD are far more rare than adult-onset MDD, with a prevalence in the general population of less than 1% (Kessler et al., 2001); with much lower rates of childhood MDD (before puberty) than adolescent-onset MDD (Green et al., 2005). Consequently, independent genetic factors may impinge upon risk for early-onset MDD relative to adult-onset MDD, which has been supported by recent research (Power et al., 2016). Our results suggest that a genetic predisposition to advanced biological ageing may shorten the time it takes for the disease to present itself, and therefore lowers the age of onset of recurrent MDD, evoking childhood-onset symptoms. Due to the fact that rs10936599 lies upstream of the gene encoding the telomerase enzyme, a discrete and modifiable biological factor, our results suggest that increasing the activity of telomerase may hinder the early onset of childhood MDD amongst those at high risk, e.g. those with a familial risk of MDD and carriers of the T-allele of rs10936599. Previous research suggests this could be achieved pharmacologically (e.g. via alterations to the immune system) or through changes to lifestyle factors (Akiyama et al., 2002, Boccardi et al., 2016).

In order to interpret the validity of our conclusions, it is important to consider whether we may have violated any of the assumptions surrounding Mendelian randomisation. The first assumption states that the genotype in question is associated with the phenotype, or biological correlate of interest (Glymour et al., 2012). This assumption was met, since we found that the rs10936599 genotype is significantly associated with shorter TL. Secondly, it is assumed that there are no unmeasured common causes of rs10936599 genotype and recurrent MDD (Glymour et al., 2012). Since no other link was found to influence both the rs10936599 genotype and childhood-onset MDD, the second assumption was also met in this study. The final assumption of Mendelian randomisation is that the instrumental variable (rs10936599 polymorphism) directly affects the outcome (childhood onset MDD) only via the exposure of interest (telomere shortening; Glymour et al., 2012). Since the instrumental variable used in our analysis was reliably associated with telomere shortening in previous studies as well as our own, and has functionally plausible effects on telomerase, an enzyme which quite specifically affects cellular ageing, this assumption was met in our study. Nonetheless, it was previously argued that the second and third assumptions are not possible to describe empirically (Martens et al., 2006). Furthermore, until understanding the fundamental biochemical mechanism relating rs10936599 genotype to childhood-onset MDD has been achieved, we need to accept the possibility that both second and third assumptions might be violated (Hung et al., 2014).

The main limitations of the current study stem from the relatively small number of childhood-onset cases, and the small-moderate predictive power of our instrumental variable. Further work will be needed to replicate our work in a larger sample to form firmer conclusions, using a more powerful instrumental variable (e.g. a polygenic risk score), as rs10936599 only explained 3% of the variance in TL in our sample. However, within the current study we do benefit from a homogenous sample set, both in terms of population structure and recurrent MDD diagnosis. Another limitation is the absence of data on maltreatment and body mass index during childhood, which may have interacted with our genetic factors to moderate risk for MDD. This would best be considered in future studies with a longitudinal study design; allowing for developmentally-sensitive gene-environment interactions to be tested.

To conclude, our study provides evidence that a genetic predisposition to advanced biological ageing may increase risk for early-onset MDD. In practice, it might be beneficial for those who are genetically vulnerable to advanced biological ageing (T-carriers of rs10936599), with a family history of MDD, to actively engage in behaviours which protect from telomere erosion, such as a healthy diet, physical activity, and the avoidance of stress (Simon et al., 2006, Richards et al., 2007, Werner et al., 2009). Future studies should further characterise the functional effect rs10936599 has on leukocyte telomerase activity, especially amongst early-onset MDD cases, and whether or not the telomerase enzyme represents an important drug target for the prevention of early-onset MDD. Further work will also be needed to understand the impact of advanced biological ageing on the developing brain and which neural mechanisms moderate risk for childhood-onset MDD.

## Funding source

TRP is funded by a Medical Research Council Skills Development Fellowship (MR/N014863/1), and the current project was funded by a Psychiatry Research Trust grant awarded to TRP and GB. The DeCC sample collection was funded by the Medical Research Council. The DeNT study was funded by Glaxo Wellcome Research and Development. The GENDEP project was supported by a European Commission Framework 6 grant (contract reference: LSHB-CT-2003-503428). GlaxoSmithKline, the Biomedical Research Centre for Mental Health at the Institute of Psychiatry, King's College London and South London, and the Maudsley National Health Service Foundation Trust (supported by the National Institute for Health Research, Department of Health, United Kingdom) provided support for add-on projects at the London recruitment centre. The Medical Research Council and GlaxoSmithKline (G0701420) provided support for genotyping within GENDEP. SELCoH was supported by the Biomedical Research Nucleus data management and informatics facility at South London and Maudsley NHS Foundation Trust, which is funded by the National Institute for Health Research (NIHR) Mental Health Biomedical Research Centre at South London and Maudsley NHS Foundation Trust and King's College London and a joint infrastructure grant from Guy's and St Thomas’ Charity and the Maudsley Charity. Phase 3 of the SELCoH study was also funded by the Maudsley Charity. MH, SLH, SF, LG and GB are supported by the National Institute for Health Research (NIHR) Mental Health Biomedical Research Centre, South London and Maudsley NHS Foundation Trust and King's College London. The views expressed are those of the authors and not necessarily those of the NHS, the NIHR or the Department of Health. The funding sources had no role in the study the design, in the collection, analysis, and interpretation of data, in the writing of the report and in the decision to submit the article for publication.

## Conflict of interest

GB has acted as a consultant in preclinical genomics and has received grants from Eli Lilly. All other authors report no conflicts of interest.
