# Supplementary material for: Genetic predisposition to advanced biological ageing increases risk for childhood-onset recurrent major depressive disorder in a large UK sample
Source: J Affect Disord. 2017 Apr 15;213:207–13. doi: 10.1016/j.jad.2017.01.017 (PMC6191533; doi:10.1016/j.jad.2017.01.017)
Supplement: Supplementary file 1 — Supplementary material [file mmc1.docx]

**Supplementary Information**

Generating Telomere Length data

To assess relative telomere length (RTL) of the 180 subject samples, two separate quantitative polymerase chain reactions (qPCRs) were performed on the ABI Prism 7900HT Sequence Detection System, with SDS software Version 2.3 used to generate the outputs. In the first reaction, we assayed the telomere repeat region (TTAGGG). In the second qPCR, we assayed a single copy gene (albumin), which we used as an internal control to correct for minor differences in DNA concentration across samples (Cawthon, 2009). Both reactions were performed across 384 well plates with identical sample/well positions used. To detect for any DNA contamination, each plate contained four negative controls (RNase-free water) in triplicate. An eight-point genomic DNA (human leukocyte) dilution series (0.47 ng, 0.94 ng, 1.88 ng, 3.75 ng, 7.5 ng, 15 ng, 30 ng, 60 ng) was also included on every plate allowing for absolute quantification of each sample, and accounting for any differences in PCR efficiency between the telomere and albumin reactions. Inter-plate variability was controlled for using five calibrator samples included on each plate, consisting of leukocyte DNA from five additional human samples, and run in triplicate. To further reduce variability between runs, the calibrators and standard curve DNA (highest standard) were both prepared in one batch and aliquoted before being frozen at -20°C. Both the calibrators and standard curve aliquots were thawed on the day of each run, with the dilution series prepared fresh on the day.

An adapted version of a qPCR protocol described in a previous paper by Cawthorn was used for each reaction (Cawthon, 2009). Each qPCR mix for the telomere reactions consisted of 10.5 uL of 2x qPCR Mastermix with SYBR green (Primer Design, Southampton, UK), 4.5 uL of RNase free water, 12ng of DNA, 1000nM of telg, 5’-ACACTAAGGTTTGGGTTTGGGTTTGGGTTTGGGTTAGTGT-3’ and 800nM of telc, 5’-TGTTAGGTATCCCTATCCCTATCCCTATCCCTATCCCTAACA-3’. Four stages made up the thermocycling conditions as follows: Stage 1: 95°C for 15 minutes, Stage 2: 2 cycles for 15 seconds at 94°C and 49°C, Stage 3: 25 cycles at 94°C for 15 seconds, 10 seconds at 62°C, and 15 seconds at 73°C (data collection), Stage 4: dissociation curve (primer specificity detection).

The same reagents and quantities as the telomere qPCR mix were used for the albumin reactions with the exception of the forward and reverse primers that were replaced with primers for the albumin gene. The quantity of both the forward and reverse primers was adjusted for the albumin reaction to 765nM of forward primer (albu): 5’-CGGCGGCGGGCGGCGCGGGCTGGGCGGAAATGCTGCACAGAATCCTT-3’ and 930nM of reverse primer (albd): 5’-GCCCGGCCCGCCGCGCCCGTCCCGCCGGAAAAGCATGGTCGCCTGTT-3’. The thermocycling conditions for the albumin reaction also consisted of four stages as follows: Stage 1: 95°C for 15 minutes, Stage 2: 2 cycles for 15 seconds at 94°C and 49°C, Stage 3: 33 cycles at 94°C for 15 seconds, 10 seconds at 62°C, and 15 seconds at 88°C (data collection), Stage 4: dissociation curve (primer specificity detection).

Calculating Relative Telomere Length

A standard deviation of <0.5 was required for at least two of the three cycle threshold (C*_t_*) technical triplicates for a sample to be included in downstream analysis. C*_q_* values were then created from the remaining C*_t_* values by relating them to absolute quantities as part of a standard curve. To adjust for inter-plate variability, mean adjusted C*_q_* values were then created by dividing the mean C*_q_* of each sample by the mean C*_q_* of the five calibrator samples (present on each plate). The RTL was then calculated by dividing each sample’s mean adjusted C*_q_* value from the telomere reaction by each sample’s mean adjusted C*_q_* values from the albumin reaction. RTL was then log-transformed to allow for parametric analysis, and adjusted for the effects of age and gender.

References

Cawthon, RM (2009) Telomere length measurement by a novel monochrome multiplex quantitative PCR method. *Nucleic Acids Res* 37: e21.
